# Wisdom of crowds in computational biology

**DOI:** 10.1371/journal.pcbi.1007032

**Published:** 2019-05-23

**Authors:** Jason A. Papin, Feilim Mac Gabhann

**Affiliations:** 1 University of Virginia, Charlottesville, Virginia, United States of America; 2 Johns Hopkins University, Baltimore, Maryland, United States of America

Scientific advances are frequently catalyzed by exploring the intersection of disciplines. From its inception, *PLOS Computational Biology* has published key insights that advance our understanding of biology and medicine—advances enabled by developments in computation and quantitative analyses. As an example, a recent initiative across the journals *PLOS Medicine*, *PLOS ONE*, and *PLOS Computational Biology* [1] resulted in a fantastic collection of research in *Machine Learning in Health and Biomedicine*. The breadth of research published in *PLOS Computational Biology* at this intersection of machine learning and health and biology was incredible, from the use of machine learning analysis to delineate biomarkers for soft tissue sarcomas [2] to the prediction of antibiotic resistance in *Escherichia coli* from pan-genome data [3]. We’re only beginning to see the power of machine learning applied to health and biology, with the hope of identifying patterns in the biological and clinical data that will lead to biomarkers of disease and the development of new clinical intervention strategies. As these data-driven strategies evolve and mature, they may also lead to a richer understanding of biological mechanisms, enabling models to predict the outcomes of scenarios and perturbations beyond the bounds of previous studies and data.

Many more cross-journal initiatives are in the works, exploring how disparate disciplines can be brought together to tackle seemingly intransigent problems with unique perspectives. Just launched is a *Targeted Anticancer Therapies and Precision Medicine* call for papers jointly with *PLOS ONE* and *PLOS Computational Biology* [4]. With nearly 10 million people dying from cancer in 2018 [5] and an increasing appreciation of the heterogeneity of the disease [6], there is an urgent need to develop targeted therapies that can be dosed, scheduled, delivered, and combined in ways that are specific to the patient. Computational approaches to this precision medicine challenge can serve as the common framework that integrates the disparate fields of expertise needed to understand the biological, pharmacological, and physiological complexity of the system. Computation can link, leverage, and amplify expertise in all the areas that are needed to understand biology and medicine: molecular dynamics, biochemistry, cell biology, human physiology, pharmacometrics, clinical practice, and more. These interdisciplinary links enable us to predict protein structure changes from gene variant data, to predict pharmacodynamics of associated drug compounds, to identify correlations in data, and simply to handle and process the vast amounts of data.

As we do in these scientific ventures, so we do in managing the activity of *PLOS Computational Biology*. This journal, like the other PLOS community journals, is led by more than 160 practicing scientists with a variety of disciplinary backgrounds. All papers submitted to the journal are evaluated by multiple scientists through the peer review process and by teams of scientists at the editorial stage. It is with this integration of these different opinions of scientists from different subdisciplines of computational biology that we try to identify and support the development and publication of outstanding science. This journal thus embraces the wisdom of crowds in the practice of science [7] as the field of computational biology develops.

*PLOS Computational Biology* is led by a “wisdom of crowds” structure, with co-editors in chief, a deputy editor-in-chief, deputy editors, associate editors, and an advisory board all bringing different perspectives to advance the field. Ruth Nussinov has been a leader of this journal for many years, recently leading as a co-editor in chief. She has been a pioneer in computational biology, recently recognized with the 2018 International Society for Computational Biology (ISCB) Accomplishment by a Senior Scientist Award [8]. Ruth will be taking on a new role with the journal; as a former editor in chief, she will oversee new journal initiatives and continue to help lead submissions through the peer review process. We thank Ruth for her exemplary and visionary leadership of the journal—past, present, and future. After serving for years as an associate editor and deputy editor for the journal, Feilim Mac Gabhann will be stepping into the co-editor in chief position. These shared leadership responsibilities across all the scientists associated with this community journal facilitate a collective wisdom as we help move the journal forward to shepherd and publish the very best research in computational biology. With greater intersection of various disciplines and opinions, we can strengthen the relationship of the community with its journal.
